# Molecular Beam-Thermal Desorption Spectrometry (MB-TDS) Monitoring of Hydrogen Desorbed from Storage Fuel Cell Anodes

**DOI:** 10.3390/ma5020248

**Published:** 2012-02-06

**Authors:** Rui F. M. Lobo, Diogo M. F. Santos, Cesar A. C. Sequeira, Jorge H. F. Ribeiro

**Affiliations:** 1 ICEMS, IST/UTL, Av. Rovisco Pais, Lisboa 1049-001, Portugal; E-Mails: diogo.santos@ist.utl.pt (D.M.F.S.); cesarseq@ist.utl.pt (C.A.C.S.); jribeiro@fct.unl.pt (J.H.F.R.); 2 Nanoscale Science Group (GNCN), Physics Department, FCT/UNL, Caparica 2829-516, Portugal

**Keywords:** hydride electrode, hydrogen storage, fuel cell anode, thermal desorption spectrometry

## Abstract

Different types of experimental studies are performed using the hydrogen storage alloy (HSA) MlNi_3.6_Co_0.85_Al_0.3_Mn_0.3_ (Ml: La-rich mischmetal), chemically surface treated, as the anode active material for application in a proton exchange membrane fuel cell (PEMFC). The recently developed molecular beam—thermal desorption spectrometry (MB-TDS) technique is here reported for detecting the electrochemical hydrogen uptake and release by the treated HSA. The MB-TDS allows an accurate determination of the hydrogen mass absorbed into the hydrogen storage alloy (HSA), and has significant advantages in comparison with the conventional TDS method. Experimental data has revealed that the membrane electrode assembly (MEA) using such chemically treated alloy presents an enhanced surface capability for hydrogen adsorption.

## 1. Introduction

Platinum has long been used as an electrocatalyst for fuel cells. However, for reasons of high cost and limited availability, the use of new electrocatalysts such as metal alloy hydrides has revealed real advantages in hydrogen storage technology. In particular, AB_5_-type metal hydride fuel cells have been widely investigated. Further studies are still welcome, though, not only regarding the development of novel nanostructured hydrogen storage materials, but also the improvement of experimental techniques for monitoring and studying the fundamental physical processes involved. The advantages of the metal hydride storage technique include safety characteristics, high volumetric storage density and low cost. Currently, many studies have confirmed the possibility of using hydrogen storage intermetallic compounds as anode active materials for application in proton exchange membrane fuel cells, instead of expensive platinum electrocatalysts. Beginning with the MlNi_3.6_Co_0.85_Al_0.3_Mn_0.3_ (Ml: La-rich mischmetal) alloy, a series of modifications including particle size reduction, surface chemical treatment and surface chemical coating, have been investigated to improve the electrochemical activity and stability of the materials in operating conditions [1,2,3,4]. Actually, intermetallic compounds are capable of reversibly absorbing large amounts of hydrogen under appropriate conditions. Charging can be done using molecular hydrogen gas or hydrogen atoms from an electrolyte [5].

This manuscript is focused on different types of experimental studies using the chemically surface treated, mixed metal alloy MlNi_3.6_Co_0.85_Al_0.3_Mn_0.3_ as the anode active material for application in a proton exchange membrane fuel cell (PEMFC). The membrane electrode assembly (MEA) is the core part of a PEMFC and the key component that controls its performance, energy distribution density and operating life. In order to enlarge the three-dimension reaction zone, extending the active material far from the electrode/electrolyte interface, the contact area between the electronically conductive hydrogen storage alloy (HSA) and the ionically conductive polymer electrolyte were maximized.

Experimental procedures with the membrane electrode assembly (MEA) using such chemically treated alloy were performed in order to improve the surface capability for hydrogen adsorption.

The reported methodology also uses the previously developed molecular-beam thermal desorption mass spectrometry (MB-TDS) [6] to detect the electrochemical hydrogen uptake and release by the chemically surface treated HSA. Actually, the MB-TDS technique can be effectively used to detect very small amounts of hydrogen which are below the detection limit of commonly used microbalances. Moreover, it has several advantages in comparison with the conventional TDS method: real time and *in-situ* detection of small amounts of hydrogen, with no requirement for previous calibration with a chemical standard [6].

## 2. Experimental Section

### 2.1. A Brief Description of MB-TDS

Molecular-beam thermal desorption mass spectrometry (MB-TDS) [6] is applied to the determination of electrochemical hydrogen uptake and release by the chemically surface treated HSA. A composite molecular beam of known intensity is produced through an orifice of known geometry, from the degassing solid sample at a certain temperature inside the oven [6].

**Figure 1:**
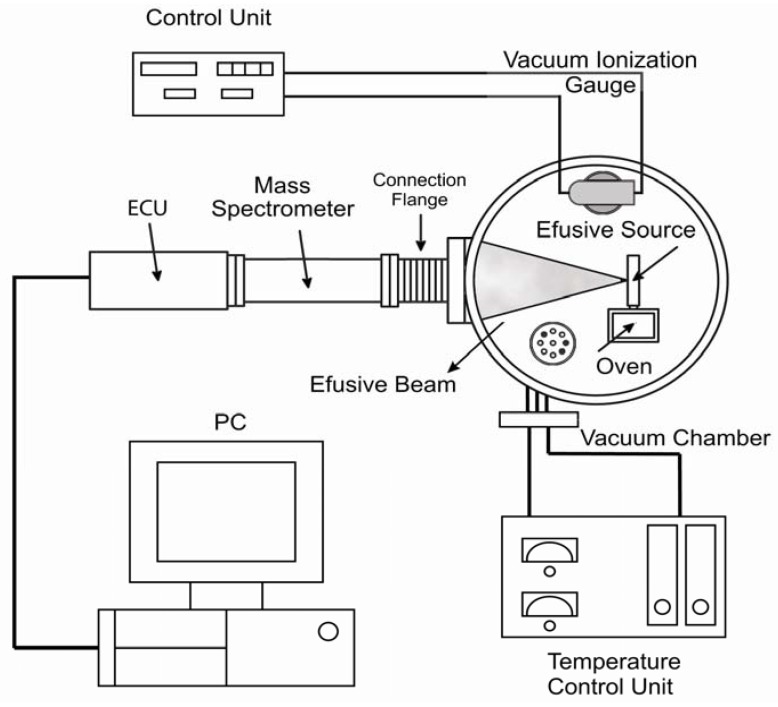
Molecular beam—thermal desorption mass spectrometer (MB-TDS) (Adapted from [6]).

The MB-TDS apparatus has already been described elsewhere [6] and is merely schematically displayed in Figure 1.

A high vacuum chamber with an appropriate pumping system hosts a quadrupole mass spectrometer and a home-made molecular beam effusive source (where the solid sample is heated). A programmable, controlled heated system will ensure that the computer records the partial pressures as a function of temperature and time. By tuning the mass spectrometer to the hydrogen mass, one can monitor the hydrogen evolution in time, thus measuring the total amount of hydrogen desorbed from the sample.

In the MB-TDS technique a molecular beam is produced by effusion through a small slit of known geometry onto the high vacuum chamber. Most commonly, the molecules comprising the beam are at a low density; that is, they are far enough apart to move independently of each other. Because of the one-directional motion of the atoms or molecules, the beam can be directed onto the mass spectrometer detector. The result is a beam of particles moving at approximately equal velocities, with few collisions occurring between them.

The effusion slit is mounted in a small gas chamber connected to the oven where the hydrogenated sample is heated (Figure 1). The combination of low pressure conditions in the high vacuum chamber, together with the low density of the beam, ensures that no collisions take place between the molecules inside the beam and those of the residual gas. This means that the effusion beam can be geometrically defined as well as its fraction detected by the quadrupole mass spectrometer (QMS) located in the forward direction.

The residual gas and molecular beam mass spectra are registered by making use of a SRS RGA100 QMS, in a mass range from 1 Da up to 100 Da. The mass filter of the QMS also allows monitoring of the time evolution of the hydrogen partial pressure.

The effusion slit with an aperture of 1 mm^2^ is located 235 mm away from the QMS and the pressure in the vacuum chamber is monitored using a Bayard-Alpert ionization gauge. The temperatures of the oven and effusion source are controlled by two independent PID (proportional-integral-differential) Eurotherm control units to maintain a selected temperature difference between them. Usually the temperature of the beam source is kept 20 °C above the temperature of the sample oven, which is a condition sufficient to avoid condensation and subsequent obstruction of the slit. Two platinum resistive temperature sensors enable both temperatures to be measured. The PID controllers enable us to use different heating rates, but values of the order of 1 °C/min have typically been used.

The MB-TDS technique is a powerful tool to avoid misleading results originating from the residual hydrogen partial pressure background variations. By subtracting the residual hydrogen gas background (measured without the beam) from the total amount of hydrogen impinging in the quadrupole spectrometer, one obtains the real amount of hydrogen coming from the sample [6,7].

### 2.2. Preparation of Samples and Characterization

The MlNi_3.6_Co_0.85_Al_0.3_Mn_0.3_ based HSA was fabricated in a Al_2_O_3_ crucible using a RF induction furnace equipped with a vacuum system. The purity of the selected components Ni, Co, Al, Mg, Mn, Ca was higher than 99% by weight. Samples were re-melted three times to ensure high levels of homogeneity. The chemical compositions of the product were analyzed using inductively-coupled plasma spectroscopy (ICP) to be consistent with the designed compositions.

The bulk alloy ingots were first mechanically pulverized into powders with dimensions of less than 200 mesh. The powder thus prepared was introduced into a stainless steel vial together with stainless steel balls (diameter: 10 mm). The powder to ball weight ratio was 1:20. The vial was then evacuated, and then filled with three different ball-milling media including, respectively, high purity argon, hydrogen gas and ethanol, and finally tightly sealed. The samples were then ball-milled by the QM-1SP Planetary Ball Mill at a rate of 225 rpm and the ball-milling time was set as required.

Part of the milled powder was submitted to surface modifications. The modification was undertaken by immersing the powder in a 6 M KOH alkaline solution containing 0.01 M KBH_4_ at 80 °C. The weight ratio of alloy powder to treatment solution was 1 g to 5 mL and the treatment was performed for a period of 3 hours. After the treatment, the alloy powder was rinsed with distilled water and then dried in a vacuum chamber.

Pt-dispersed carbon (Pt/C) powder with a Pt content of 39% by weight (Product No. 44830, Alfa Aesar, Ward Hill, MA, USA) was employed as the electrocatalyst of the membrane electrode assembly (MEA). Nafion 115 membrane (Product No. 42179, Alfa Aesar, Ward Hill, MA, USA) and 5% by weight Nafion solution (Product No. 42117, Alfa Aesar, Ward Hill, MA, USA) were employed as PEM and proton conducting material for the impregnation, respectively. A sheet of carbon fiber paper (TGP-H-060, 190 ∞m in thickness, Toray Composites America, Inc., Ward Hill, MA, USA) was soaked in a PTFE solution for hydrophobic treatment, to be used as the MEA diffusion layer. An ink prepared by mixing the active material with PTFE and Nafion solution was sprayed onto the carbon diffusion layer. All parts including the electrodes and membrane were assembled in sequence and the MEAs were tested in a PEMFC test stand using conventional electrochemical equipment.

The measurement of the p-c-T for the hydrogen storage alloy was determined by the Sieverts method and examined on a HI-980001 type p-c-T apparatus. The alloy sample was ground to below 200 mesh in advance. The particle size, distribution and the specific surface area analysis were carried out by a Mastersizer 2000 Particle Size Analyser (PSA) from Malvern Instruments.

## 3. Results and Discussion

The study of Pt catalysts revealed that the reduction of the particle size of Pt would greatly enhance its utilization rate. N. Giordano *et al.* [8] found that when the diameter of Pt is reduced to about 3 nm, the electrocatalytic activity reaches a maximum value. Accordingly, by increasing the specific surface area of the electrode material by reducing the size of active material using the ball mill, one could expect to improve the MEA electrochemical properties.

**Figure 2:**
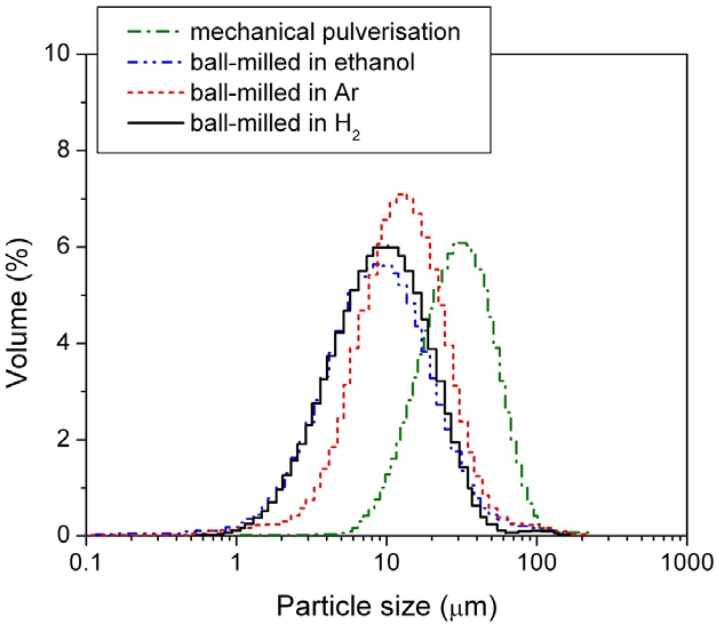
Particle size distribution of the mechanically pulverized HSA and of the HSAs ball-milled in different media.

In this study, the alloy samples were modified using three different ball-mill media, *i.e.*, under 3 atm H_2_, under 3 atm Ar, and in passivating ethanol solution. After milling, the sizes of the HSA particles were examined by the PSA. The results are shown in Figure 2 and mean particle sizes are listed in Table 1.

**Table 1:** Mean particle size and specific surface area of the mechanically pulverized hydrogen storage alloy (HSA) and of the HSAs ball-milled in different media.

Preparation method	Mean particle size (μm)	Specific surface area (m^2^·g^−1^)
Ball-milled under 3 atm H_2_	11.969	1.2069
Ball-milled under 3 atm Ar	12.272	1.1702
Ball-milled in ethanol	16.561	0.8096
Mechanical pulverisation	43.410	0.3272

Among the three different modification media, the HSA sample ball-milled in 3 atm H_2_ had the smallest mean particle size of 11.969 μm and exhibited the best concentrated distribution of particle size of HSA. During the ball milling in 3 atm H_2_, the HSA likely reacted with H_2_ to form the metal hydride. The material in the hydride phase is much more brittle compared with the corresponding dehydrided phase, resulting in easier pulverization and the smaller product size in the final ball mill.

Based on the studies of loading amount and ball mill products in different applied environments and associated with the discussion on surface modifications, an optimized HSA anode active material was prepared using a set of modifications including ball milling in H_2_ as milling media, surface modification in a hot alkaline solution containing KBH_4_ and chemical coating with Pd. It has been observed that the cell performance of the HSA anode MEA is remarkably improved.

**Figure 3:**
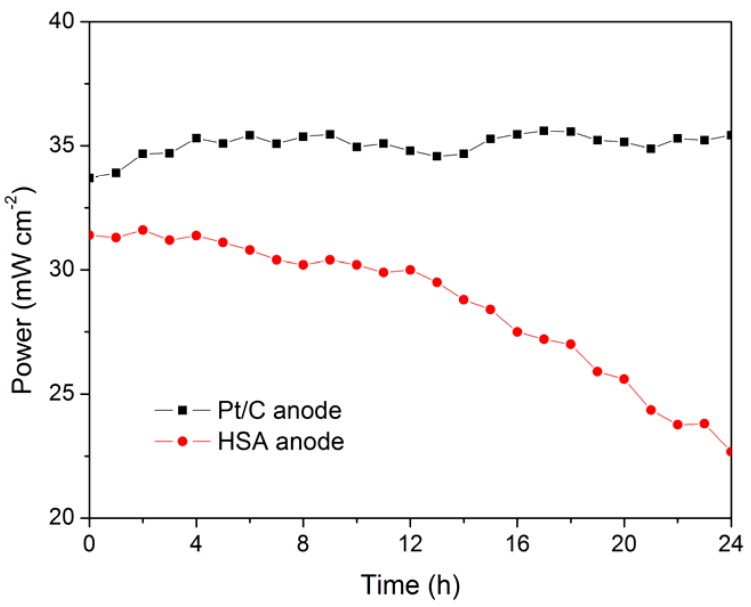
Working stabilities of the HSA anode membrane electrode assembly (MEA) in comparison with Pt/C MEA at a constant current load of 40 mA/cm^2^ (operating conditions: electrode area 1 cm^2^, pressure P_H_2__ = P_O_2__ = 2 atm; working temperature T_cell_ = 25 °C, wetting temperature T_anode_ = T_cathode_ = 40 °C).

The working stabilities of the optimized HSA anode MEA and the Pt/C anode MEA at a constant current load of 40 mA/cm^2^ were studied and compared. As shown in Figure 3, the power of the HSA anode MEA was at 22.68 mW/cm^2^ and 72.4% of its initial power density after 24 hours. No sign of degradation in Pt/C anode MEA implies that the use of HSA active material results in the performance degradation of the HSA anode MEA.

**Figure 4:**
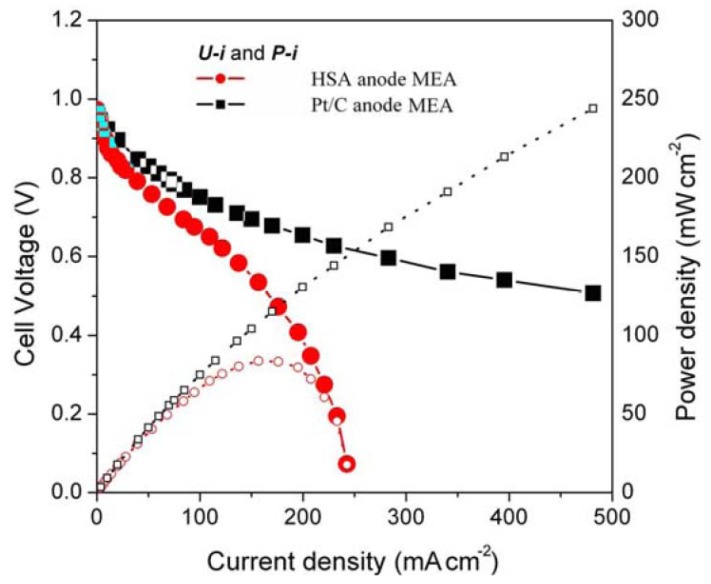
*U-i* and *P-i* characteristics for the HSA anode MEA and for the Pt/C anode MEA. Operating conditions: A = 1 cm^2^, $P_{H_{2}}$ = $P_{O_{2}}$ = 2 atm, *T*_cell_ = *T*_anode_ = *T*_cathode_ = 60 °C. Open symbols refer to the RH scale.

In addition, Figure 4 shows the HSA anode MEA electric performances and comparison with the Pt/C anode MEA.

Figure 4 illustrates the *U*-*i* and *P*-*i* characteristics of the optimized HSA anode MEA and the Pt/C MEA. It can be observed that the cell performance of the HSA anode MEA is very good. The discharge current densities of the HSA anode MEA reached 168 mA/cm^2^ at 0.5 V and 232.4 mA/cm^2^ at 0.2 V respectively, with maximum power density going up to 84 mW/cm^2^.

The hydrogen content of a sample of MlNi_3.6_Co_0.85_Al_0.3_Mn_0.3_, with hydrogen previously absorbed, was monitored by molecular beam-thermal desorption spectrometry (MB-TDS). The hydrogen pre-charging was performed galvanostatically using pure Pd as the anode of an electrolytic cell containing an aqueous 1 M KOH electrolyte, and imposing an electric current of 133 mA.

The same electrochemical hydrogen uptake procedure was applied to a new dry hydrogen storage alloy sample, keeping the physical operating parameters as before but using a much smaller time of charge to ensure that the amount of hydrogen absorbed was not high enough to be detected by the Kern microbalance.

**Figure 5:**
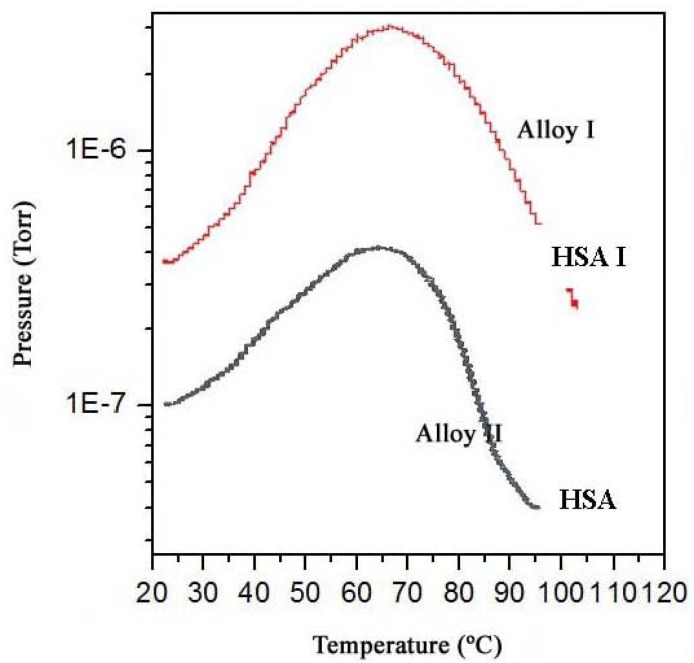
MB-TDS spectrum of two (previously electrochemical) charged hydrogenated MlNi_3.6_Co_0.85_Al_0.3_Mn_0.3_ alloys submitted to a heating rate of 1 °C/min. The HSA (I) has a larger absorbed hydrogen mass than HSA (II). Adapted from [6].

The dry HSA electrode was weighed with a Kern ABS analytical balance before and after the electrochemical hydrogen charging. On average, the weight difference was 0.7 ± 0.1 mg. The MB-TDS study of the HSA sample was performed at a heating rate of 1 °C·min^−1^. The resulting desorption spectrum is shown in Figure 5.

The total amount of hydrogen contained in the HSA sample was computed by considering the integral of the Gaussian MB-TDS spectrum displayed in Figure 4, followed by subtraction of the hydrogen background pressure taken in similar experimental conditions (*i.e.*, 1.0 × 10^−7^ Torr) [6,7].

Actually, the number of hydrogen molecules which were desorbed during the MB-TDS measurements are easily estimated from the area under the desorption curve after discounting the above-mentioned hydrogen background pressure. The result is 0.1 Pa s. This value is consistent with the hydrogen content in the sample previously weighed (0.7 mg) if the pumping speed is of the order of 3 × 10^−2^ dm^3^·s^−1^. In fact, 0.7 mg of hydrogen corresponds to about 2 × 10^20^ hydrogen molecules, and thus an average temperature of 100 °C results in a pumping speed of about 3 × 10^−2^ dm^3^·s^−1^.

However, the experimental pumping speed of the vacuum system is 10 times larger. Such a discrepancy can be attributed jointly to the geometrical fraction of the hydrogen beam effectively “seen” by the quadrupole detector and the ionization efficiency of this detector [6,7,9].

**Figure 6:**
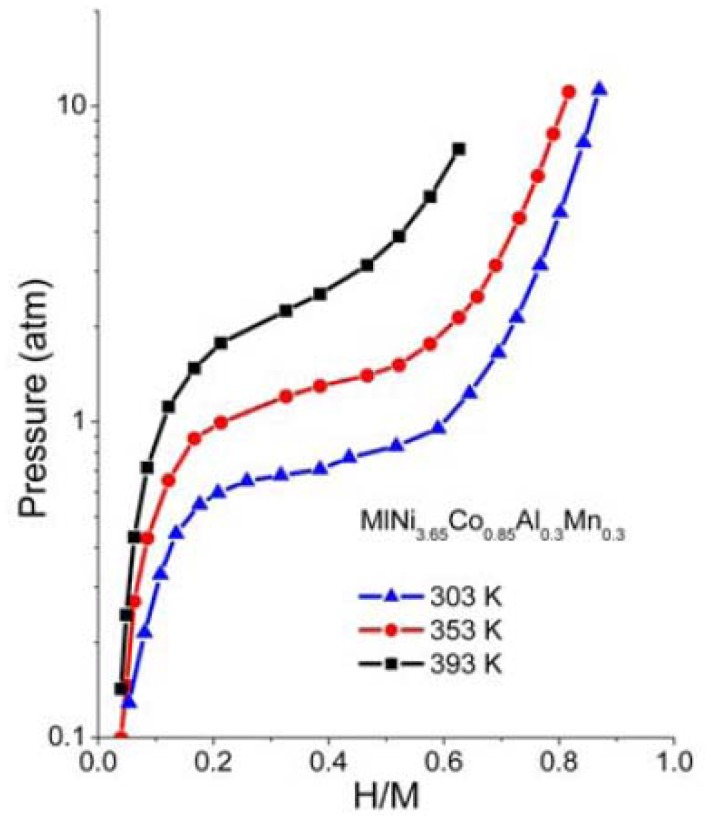
p-c-T isotherms for HSA.

The p-c-T curves of the HSA were investigated and are plotted in Figure 6 and we see a distinct hydrogen sorption plateau displayed between 300 K and 350 K.

**Figure 7:**
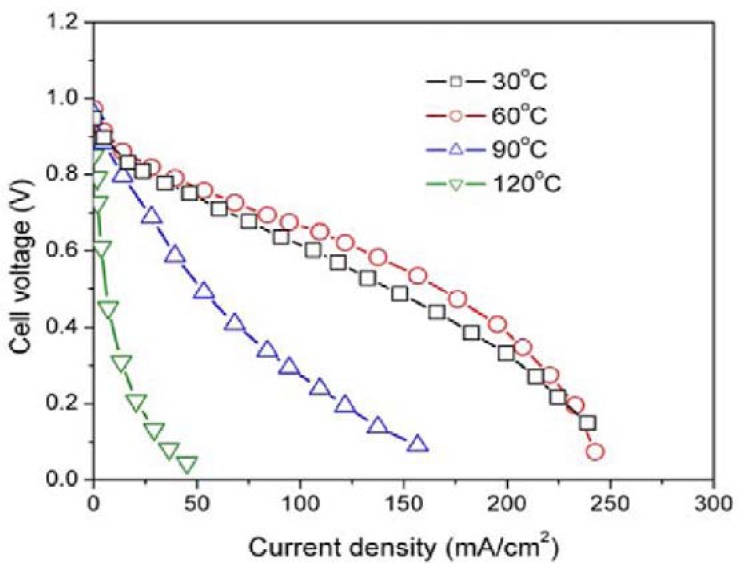
Cell performance of HSA-MEA anode at different temperatures.

Additionally, in Figure 7 the experimental results of the effect of temperature on the cell performance of the HSA anode MEA are displayed. The electrode parameters relating to four different temperature regimes were calculated and are listed in Table 2. The results indicated that HSA—MEA anode attains its optimum cell performance when the working temperature is 60 °C.

**Table 2:** Electrode parameters of MEA anode at different temperatures (assuming the anode as MEA reaction rate control electrode).

T_cell_ (°C)	U_0_ (V)	b_Tafel_ (V/dec)	R_i_(Ω/cm^2^)	i_0_ (A/cm^2^)
30	0.7828	0.02343	2.53	1.95 × 10^−4^
60	0.8071	0.02093	2.09	6.42 × 10^−4^
90	0.7592	0.03395	7.27	5.48 × 10^−4^
120	^____^	-	41.5	-

The relationship between the working temperature and the *U*-*i* characteristics and the calculated electrode parameters can be summarized as follows: with a rise in working temperature, the rate of the electrode reaction increases and the activation polarization decreases (with the *Tafel* slope *b_Tafel_* dropping from 0.02343 V/deg. at 30 °C to 0.02093 V/deg. at 60 °C), as well as the mass transfer resistance and the ohmic polarization (the value *R_i_* dropped from 2.53 Ω/cm^2^ at 30 °C to 2.09 Ω/cm^2^ at 60 °C); however, for very high temperatures (~90 °C), the cell performance dropped further and the electrocatalytic activities of the HSA also decreases, with the value of *b_Tafel_* rising from 0.02093 V/deg. at 60 °C to 0.03395 V/deg.; when the temperature attains 120 °C, the *b_Tafel_* cannot be calculated according to the actual *U*-*i* curves, and *R_i_* value quickly goes up to 41.5 Ω/cm^2^.

## 4. Conclusions

The electrochemical properties of a PEMFC MEA were examined using differently prepared HSAs instead of Pt/C as the anode active material. The results revealed that MEA using the HSA anode has a good catalytic activity and working stability. The HSA anode displayed the best performance at a working temperature was 60 °C. At temperatures lower than 60 °C the electrode reaction rate was too slow and at higher temperatures the catalytic hydride phase became unstable.

The performance of the PEMFC was improved when operating at temperatures of ~120 °C. The p-c-T plateau pressure of this HSA was shown to be unable to provide high hydrogen sorption kinetics for operating temperatures higher than 60 °C.

The total amount of hydrogen contents in the HSA sample could be computed from the MB-TDS spectrum, which offers an alternative to avoid misleading weight results in case of minimal amounts of hydrogen uptake.

MB-TDS is a powerful procedure for quantitative analysis in real time and *in-situ* for hydrogen uptake by any kind of hydrogen storage material, even when the amount is below the detection limit of a microbalance. The MB-TDS spectrum delivers a quick overview at the temperature of the desorption. In addition, to perform a quantitative analysis of the hydrogen storage capacity, the apparatus does not need to be calibrated by introducing a specific amount of hydrogen molecules into the high-vacuum system, contrary to usual practice occurs in conventional TDS.
